# Autologous adipose-derived stem cells for the treatment of Crohn’s fistula-in-ano: an open-label, controlled trial

**DOI:** 10.1186/s13287-020-01636-4

**Published:** 2020-03-17

**Authors:** Chungen Zhou, Meng Li, Yang Zhang, Min Ni, Yehuang Wang, Dachao Xu, Yang Shi, Bo Zhang, Yanni Chen, Yan Huang, Sumin Zhang, Hongzhen Shi, Bin Jiang

**Affiliations:** 1 Graduate School of Nanjing University of Chinese Medicine, Nanjing, 210029 Jiangsu Province China; 2 Colorectal Disease Center of Nanjing Hospital of Chinese Medicine Affiliated to Nanjing University of Chinese Medicine, Nanjing, 210022 Jiangsu Province China; 3 Reaserch Institute of Jiangsu Decon Bio-science Technologies Company Ltd., Nanjing, 210000 Jiangsu Province China; 4 Zhongda Hospital Southeast University, Nanjing, 210009 Jiangsu Province China; 5 Yale School of Engineering & Applied Science, New Haven, 06520-8292 Connecticut USA

**Keywords:** Autologous adipose-derived stem cells, Stem cell transplantation, Crohn’s fistula-in-ano, Efficacy, Safety

## Abstract

**Background:**

Crohn’s fistula-in-ano is a refractory disease in colorectal and anal surgery. Although autologous adipose-derived stem cell (ADSC) has been used in the treatment of Crohn’s fistula-in-ano because of its convenience, non-incision of normal tissue, good tolerance, repeatability, quick recovery, less pain, less damage to anal function, and high quality of life during the perioperative period, there are no reports of its use in China. This is the first clinical trial in China on the treatment of Crohn’s fistula-in-ano with ADSC to evaluate its efficacy and safety.

**Methods:**

A total of 22 patients with Crohn’s fistula-in-ano were enrolled in this study from January 2018 to October 2018 in the Colorectal Disease Center of Nanjing Hospital of Chinese Medicine Affiliated to Nanjing University of Chinese Medicine. Patients were divided (1:1) into an observation group (ADSC) and a control group (incision-thread-drawing procedure). Primary efficacy endpoint evaluated at months 3, 6, and 12 was the closure of fistulas (closure of all treated fistulas at baseline, confirmed by doctor’s clinical assessment and magnetic resonance imaging or transrectal ultrasonography). The patients additionally completed some scoring scales at each follow-up including simplified Crohn’s Disease Activity Index (CDAI), Perianal Disease Activity Index (PDAI), Inflammatory Bowel Disease Questionnaire (IBDQ), pain scores with visual analog score (VAS), and Wexner score. The data of inflammatory indexes were also collected.

**Results:**

The healing rates of the observation group and the control group at months 3, 6, and 12 were as follows: 10/11(90.9%) vs 5/11(45.5%), 8/11(72.7%) vs 6/11(54.5%), and 7/11(63.6%) vs 6/11(54.5%), respectively. There was no statistical difference between the two groups. In addition, the improvement in simplified CDAI, PDAI, IBDQ, VAS, and Wexner score of the observation group were better than that of the control group at each follow-up. The inflammatory indexes decreased in both the observation group and the control group at 3 months follow-up. And there were no significant differences in the changes of inflammatory indexes between two groups at month 3 compared with the baseline. Safety was maintained throughout month 12, and adverse events occurred in 63.6% of patients in the observation group and 100% patients in the control group. And no adverse event associated with ADSC injection was observed in the study.

**Conclusion:**

ADSC is a feasible and effective treatment for Crohn’s fistula-in-ano, compared with traditional incision and thread-drawing. It can protect anal function of patients, relieve pain, allow quick recovery, be well-tolerated, and improve the quality of life during perioperative period.

**Trial registration:**

China Clinical Trials Registry, No. ChiCTR1800014599. Registered 23 January 2018.

## Introduction

Crohn’s disease is a lifelong chronic nonspecific intestinal inflammatory disease. Its etiology and pathogenesis are not yet clear [1, 2]. Perianal fistulas, especially complex anal fistula, are recognized complications of Crohn’s disease (CD), which can lead to substantial morbidity and reduce quality of life [3]. Complex anal fistula, especially Crohn’s fistula-in-ano, is a refractory disease in colorectal and anal surgery [4]. In addition to medical treatments, surgery is an indispensable part of the treatment for Crohn’s fistula-in-ano, especially drainage [5]. However, traditional surgical procedures require cutting normal tissues that injure the anal sphincter in varying degrees and cause large drainage wounds, severe pain, and slow healing [4–6]. Therefore, surgery for anal fistula should be minimally invasive [7]. Among the minimally invasive procedures used, adipose-derived stem cell (ADSC) transplantation is a research hotspot. At present, ADSCs have been used in inflammatory bowel disease (IBD), osteoarthritis, bone repair, cardiovascular disease, diabetes, nervous system disease, plastic surgery, idiopathic pulmonary fibrosis, chronic liver injury, acute renal injury, and so on [8]. In the treatment of IBD, many researches have achieved good results in the treatment of Crohn’s fistula-in-ano, but there is not one in China. This article introduces the first clinical trial of autologous ADSCs in the treatment of Crohn’s fistula-in-ano in China.

## Materials and methods

### Study population

A total of 22 patients in Nanjing Hospital of Chinese Medicine were recruited and enrolled in the study from January 2018 to October 2018. Their age ranged from 12 to 51 years old, with the average age being 28.86 ± 10.13 years old. Only one of them is female and they all signed informed consent before enrollment.

Inclusion criteria are as follows: ① diagnosis of complex Crohn’s fistula-in-ano, which meet the criteria of clinical practice guideline for the management of anorectal abscess, fistula-in-ano, and rectovaginal that was published by American Society of Colon and Rectal Surgeons in 2016. ② Patients with Crohn’s disease should control their disease in remission or mild active phase, that is, simplified Crohn’s Disease Activity Index (CDAI) is less than 6 points. ③ There is no evidence of cancer or precancerous lesions in enteroscopy 1 year before admission. ④ There is no other cardio-cerebrovascular diseases.

Exclusion criteria are as follows: ① acute infection stage of anal fistula (immature fistula). ② Patients with Crohn’s disease’s simplified CDAI > 6. ③ An autoimmune disease other than Crohn’s disease. ④ Patients with infectious diseases. ⑤ Patients who were allergic to anesthetics. ⑥ Patients who cannot tolerate liposuction. ⑦ Patients who were pregnant or were trying to become pregnant.

### Study protocol

The study was conducted according to the principles of the Declaration of Helsinki. And the protocol was approved by the Ethics Review Committee of Nanjing Hospital of Chinese Medicine (Ethics Review No. KY2018011), and registered with the China Clinical Trials Registry (No. ChiCTR1800014599).

**Fig. 1:**
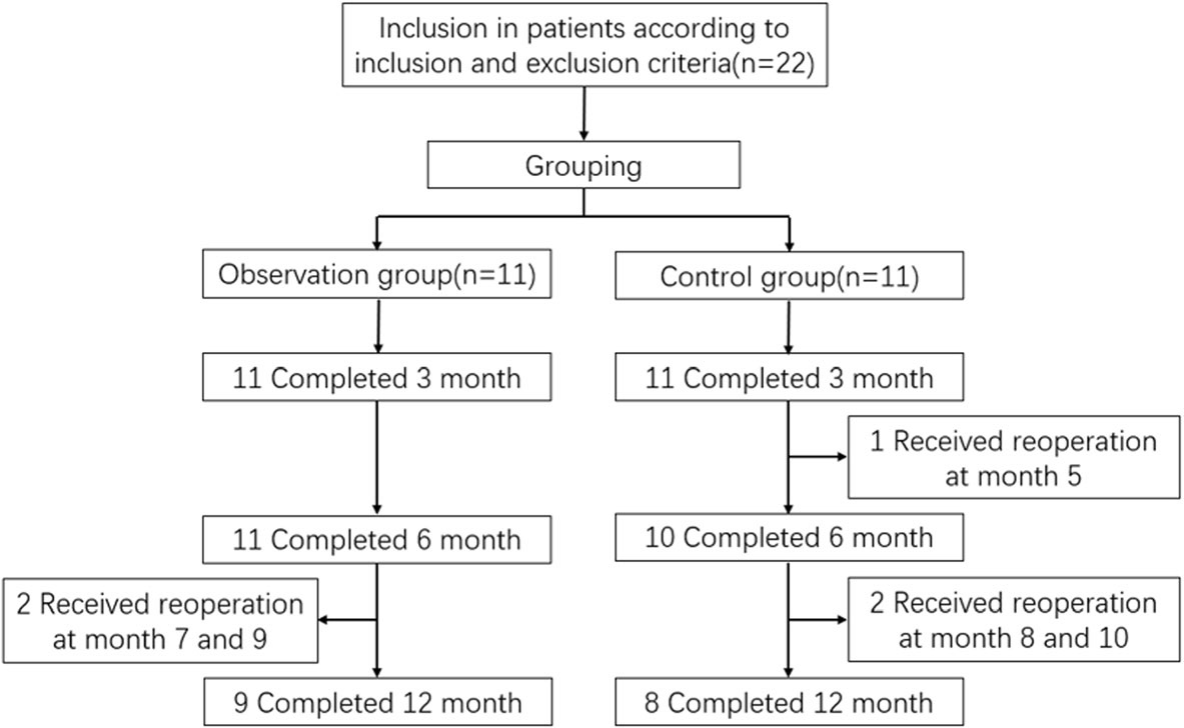
Flow chart of the study

This study was designed as an open-label, randomized, controlled clinical trial to evaluate the efficacy and safety of ADSCs in the treatment of Crohn’s fistula-in-ano. The patients were divided into an observation group (ADSCs) and control group (incision-thread-drawing procedure). Patients in the control group received the traditional treatment of incision-thread-drawing procedure, while patients in the observation group received ADSC treatment. The specific treatment process is as follows: every patient was followed up for a minimum of 24 weeks to evaluate the efficacy and safety of ADSC treatment. Figure 1 shows the flow chart of the study.

#### Fistula preparation

After admission, patients received fistula preparation more than 2 weeks before ADSC injection, which included fistula exploration, curettage, and drainage with seton.

#### Preparation of ADSCs

**Fig. 2:**
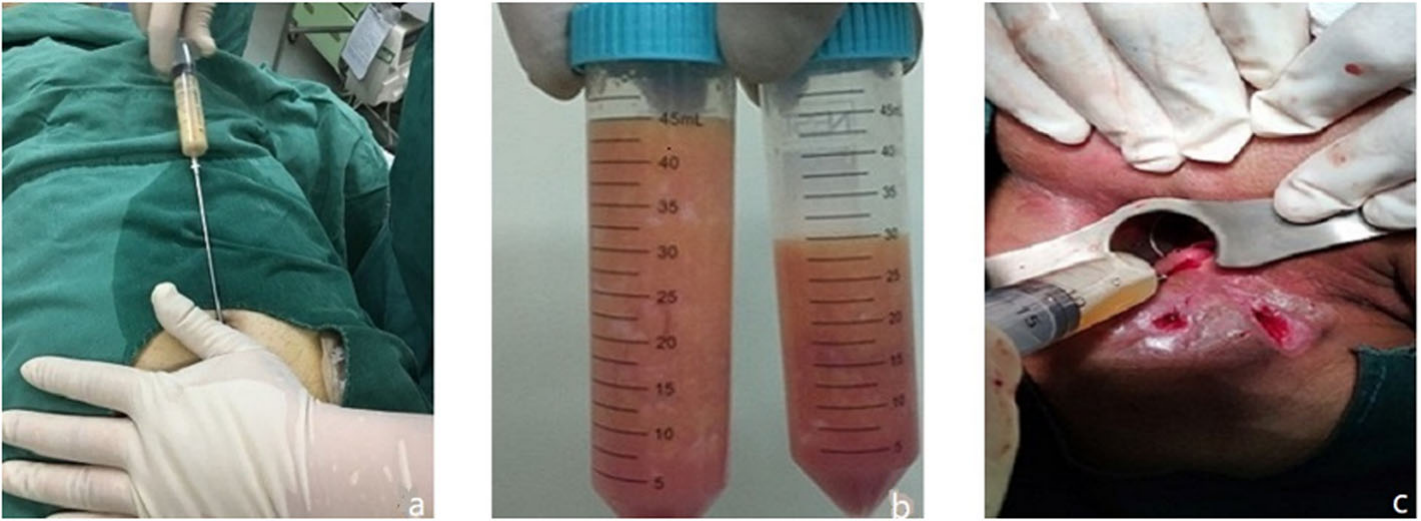
Liposuction and injection. **a** Liposuction. **b** Fat of the patient. **c** Injection of ADSCs

**Fig. 3:**
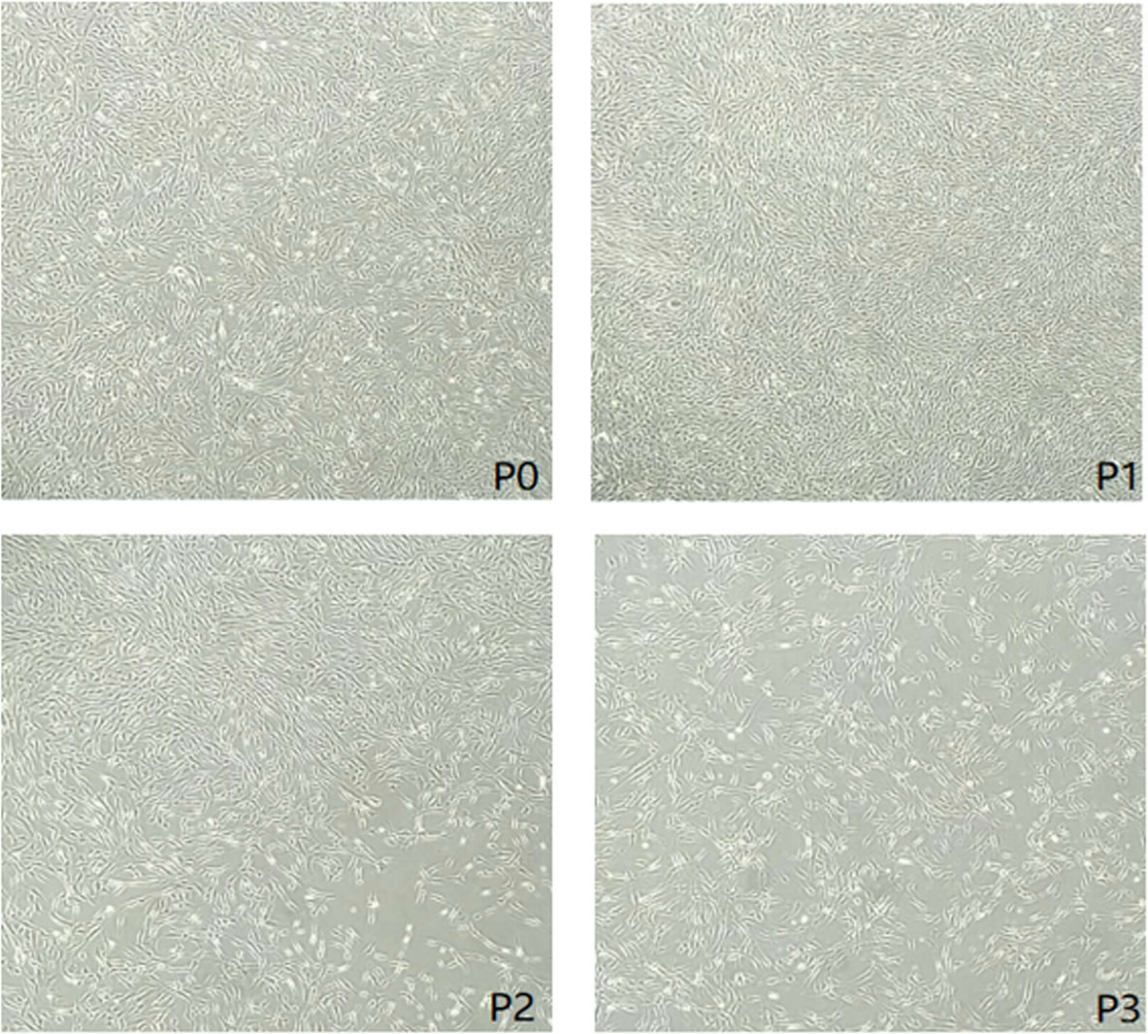
Cell morphology of each generation

**Fig. 4:**
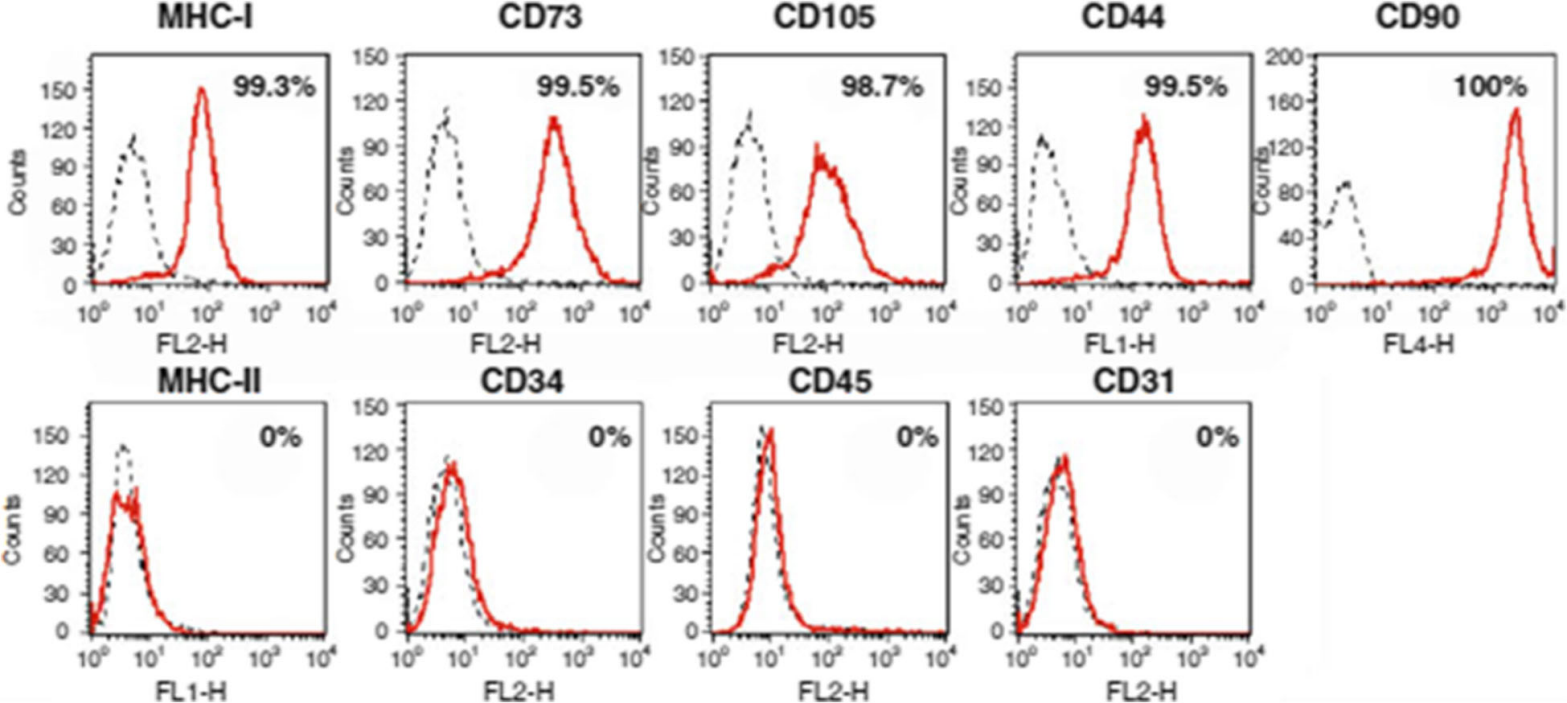
Cell phenotype of ADSCs

Liposuction from the abdomen and thighs was performed on patients in the observation group (Fig. 2a, b). Then the fat was separated, cultured, proliferated, and identified as follows: First, the adipose tissue was washed with sterile normal saline, and then the corresponding concentration of collagenase I was added. After 60 min of shaking digestion at 37 °C, the upper lipid and liquid layers were centrifuged and absorbed, and the cells were re-suspended in normal saline and filtered by cell filters. The stromal vascular fraction (SVF) was obtained by removing the undigested tissue, centrifuging the filtrate, and discarding the supernatant. Trypan blue staining was used to count the number and activity of cells. SVF was then inoculated into culture flask. Serum-free medium and serum substitutes were added. The culture conditions were 5% CO_2_ and 37 °C. When the degree of cell fusion reached 70~80%, trypsin was added to digest the cells, and the digested cell suspension was collected and inoculated into the culture flask for subculture. ADSCs of the third or fourth generation were collected to detect cell morphology, viability, cell phenotype, endotoxin, bacteria, fungi, and mycoplasma (Figs. 3 and 4). After identification, it was frozen at − 80 °C, thawed and resuscitated on the day of injection, and transported to the operating room at 15–25 °C.

#### Injection procedure

More than 2 weeks after fistula preparation, patients in the observation group received injections with ADSCs. First, the fistula and its internal and external orifices were carefully explored with probes to avoid missing branches and pus cavities. After the exploration, the epithelial tissue of the fistula was destroyed from inside to outside by metal brush and electrocoagulation (COOK MEDICAL, USA, Registered Import of National Equipment 20153462399), and the necrotic tissue of the fistula wall was removed by washing with distilled water. After cleaning, the inner opening was closed with 2-0 vicryl. ADSC suspension containing 5 × 10^6^cells/ml was injected uniformly into the inner orifice and around the fistula wall with a syringe (Fig. 2c). Multiple injections (> 4 times) were carried out in all quadrants. Finally, serum suspension containing 1 × 10^6^ cells/ml was perfused into the fistula and the external opening was closed.

The dosage of ADSCs is based on the diameter and length of fistula measured before injection, and mainly according to the results of preoperative MRI and clinical evaluation at fistula preparation. The diameter of the fistula was less than 1 cm, and 1 ml ADSCs/cm was injected into the fistula. And 2 ml ADSCs/cm was injected into the fistula in the patients with the fistula diameter ranging from 1 and 2 cm.

### Assessments

#### Evaluation of efficacy

**Fig. 5:**
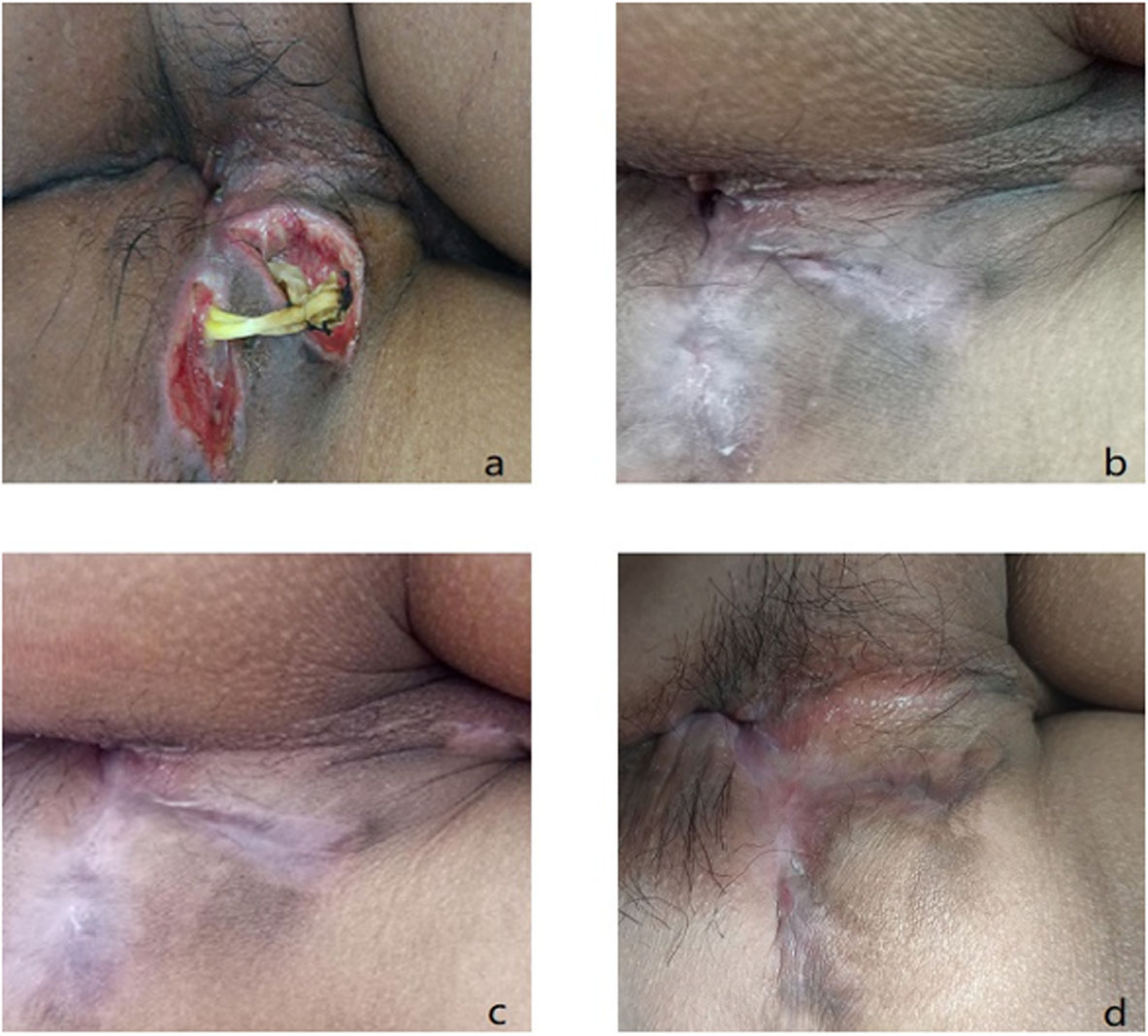
A representative case showing a high intersphincteric fistula resulted in Crohn’s disease. A 25-year-old male patient had been diagnosed with an anal fistula in July 2017 and was assigned to observation group. This patient was completely healed at 3 months and complete healing was sustained for up to 12 months after injection. **a** Preinjection, **b** 3 months, **c** 6 months, and **d** 12 months after injection of ADSCs

The primary end point for efficacy was defined as the proportion of patients whose fistula had healed at months 3, 6, and 12 postoperatively. The researchers evaluated healing of the fistula through clinical evaluation at each follow-up (Fig. 2) and by magnetic resonance imaging (MRI) or endorectal ultrasonography (ERUS) at 3, 6, and 12 months postoperatively (Fig. 5). Healing was defined as the complete epithelialization of external openings (i.e., no pus outflow from the external openings under any circumstances) and no evidence of fistulas in MRI or ERUS.

The secondary end points for efficacy included simplified CDAI, Perianal Disease Activity Index (PDAI), Inflammatory Bowel Disease Questionnaire (IBDQ), pain scores with visual analog score (VAS), and Wexner score. And the patients completed these tests at each follow-up. In addition, we also recorded the inflammatory indexes of patients at baseline and month 3 postoperatively including high sensitivity C-reactive protein (CRP), erythrocyte sedimentation rate (ESR), and fecal calprotectin (FC).

#### Evaluation of safety

Safety was assessed by determining the incidence of adverse events (AEs) and serious AEs. During each follow-up, the AEs of patients were monitored. Perianal areas were assessed for the following: formation of abnormal tissue, persistence of or increased signs of inflammation, and any other observations that might indicate the impact of ADSCs.

### Statistical analyses

The researchers used SPSS 22.0 software to process data. The last observation carried forward (LOCF) approach was applied in the case of missing data. Mean values were used to describe the measurement data, and *t* test or rank sum test were used for analysis. The counting data were described by frequency (constituent ratio) and analyzed by *X*^2^ test or Fisher exact test. *P* < 0.05 was considered statistically significant.

## Results

### Study population

Twenty-two patients were screened and were assigned into two groups: 11 to observation group and 11 to control group (Fig. 1). The baseline characteristics of the two groups were similar (Table 1). All patients completed the 3-month and 6-month follow-up but only 17 patients completed 12-month follow-up because another five received reoperation due to the recurrence and no healing of fistulas (Fig. 1). During the study, all patients received aminosalicylic acid (Mesalazine) and probiotic treatment. One patient in the observation group and three patients in the control group received immunomodulator treatment. One patient in each group was given antibiotics. No patient received anti-TNF or glucocorticoid treatment in the study. The average volume of fat extracted from the patients in the observation was 48.4 ± 23.1 mL and number of ADSC was (142.3 ± 45.7) × 10^6^cells.

**Table 1:** Baseline data

**Contents**	**Observation group (*n*** **= 11)**	**Control group (*n*** **= 11)**
**Sex**		
Male	11 (100.0%)	10 (90.9%)
Female	0 (0.0%)	1 (9.1%)
**Age (year)**	24.4 ± 5.0	24.9 ± 5.4
**BMI (kg/m**^**2**^**)**	21.8 ± 3.0	19.9 ± 2.6
**Volume of fat (mL)**	48.4 ± 23.1	–
**Number of cells (×10**^**6**^**cells)**	142.3 ± 45.7	–
**Simplified CDAI**^†^	4.3 ± 0.5	4.4 ± 0.7
PDAI^*^	8.5 ± 1.4	9.5 ± 1.7
**IBDQ**^‡^	143.5 ± 19.8	137.9 ± 29.7
**VAS**^**§**^	0.8 ± 1.0	1.1 ± 1.0
**Wexner**^**¶**^	1.2 ± 1.3	1.5 ± 0.7
**Location of Crohn’s disease**		
Ileum (L1)	1 (9.1%)	1 (9.1%)
Colon (L2)	4 (36.4%)	3 (27.3%)
Ileocolon (L3)	6 (54.6%)	7 (63.6%)
Upper gastrointestinal (L4)	0 (0.0%)	0 (0.0%)
**Classification of fistula-in-ano (Parks)**		
Transsphincteric	4 (36.4%)	7 (63.6%)
Suprasphincteric	1 (9.1%)	0 (0.0%)
Intersphincteric	4 (36.4%)	3 (27.3%)
Extrasphincteric	2 (18.2%)	1 (9.1%)
**Number of internal openings**		
1	9 (81.8%)	10 (90.9%)
2	2 (18.2%)	1 (9.1%)
**Number of external openings**		
1	5 (45.4%)	5 (45.4%)
2	3 (27.3%)	4 (36.4%)
3	2 (18.2%)	2 (18.2%)
4	1 (9.1%)	0 (0.0%)
**Drug use at baseline**		
ASA	11/11 (100.0%)	11/11 (100.0%)
Probiotics	11/11 (100.0%)	11/11 (100.0%)
Immunomodulators	1/11 (9.1%)	3/11 (27.3%)
Antibiotics	1/11 (9.1%)	1/11 (9.1%)
Anti-TNF	0/11 (0.0%)	0/11 (0.0%)
Glucocorticoids	0/11 (0.0%)	0/11 (0.0%)

Data are mean (SD) or number (%). Percentages might not always add up to exactly 100% as a result of rounding. BMI = body mass index. ASA = aminosalicylic acid. TNF = tumor necrosis factor. †Scores Less than 4 is divided into remission period, 5–8 is divided into moderate active period, and more than 9 is divided into severe active period. *Scores range from 0 to 20; higher scores suggest more severe disease. ‡Scores range from 32 to 224; higher scores suggest better quality of life. §Scores range from 0 to 10; higher scores suggest more severe degree of pain. ¶Scores range from 0 to 20; higher scores suggest more severe degree of anal incontinence

### Efficacy

#### Primary end point

**Fig. 6:**
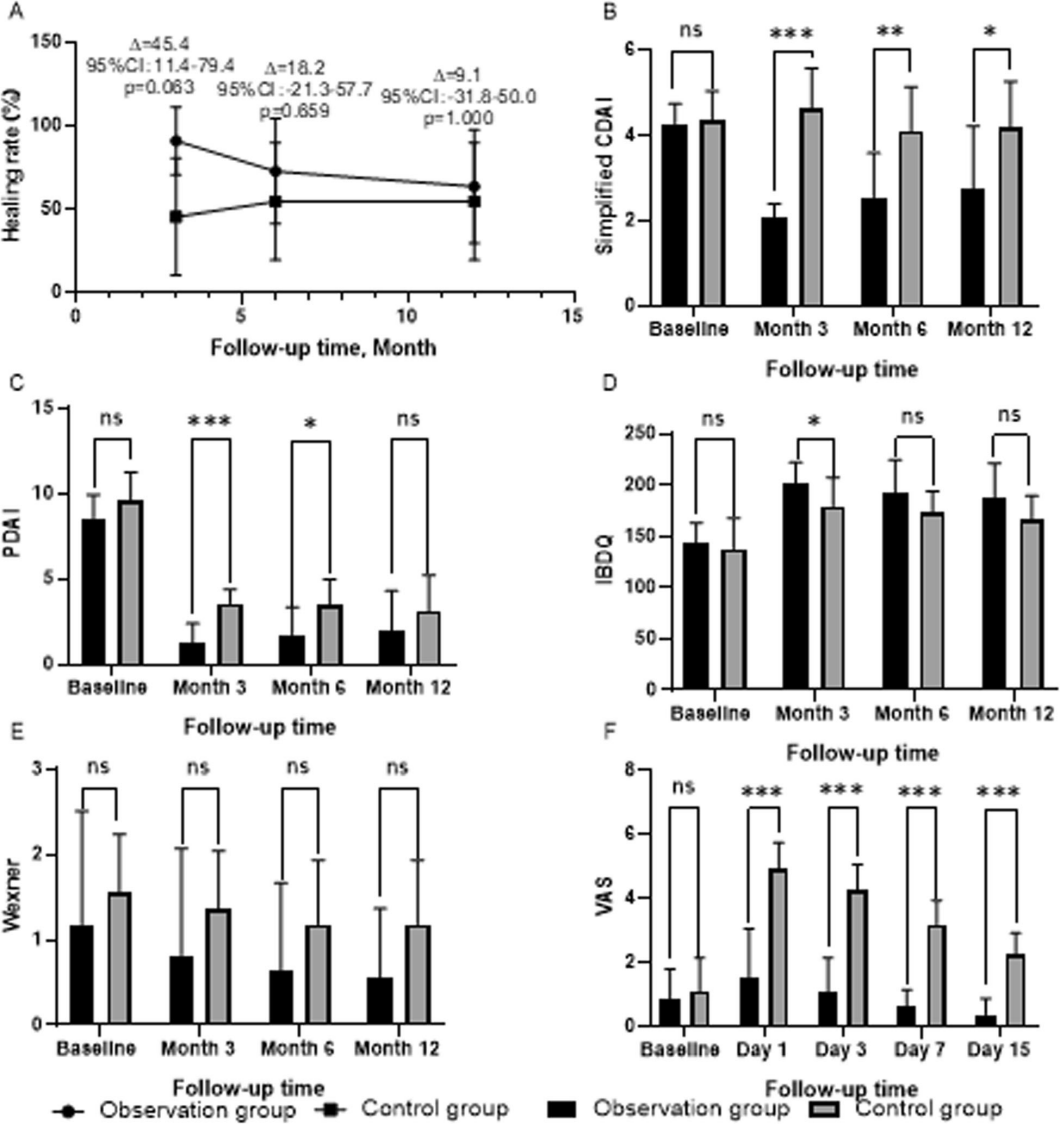
End points. **a** Fistulas healing rate of patients, **b** Simplified CDAI score of patients, **c** PDAI score of patients, **d** IBDQ score of patients, **e** Wexner score of patients, **f** VAS score of patients; ∆ = Absolute value of difference in healing rate between two groups; ns = no significance, ✱✱✱ = *p* < 0.001, ✱✱ = *p* < 0.01, ✱ = *p* < 0.05

At month 3, there were no significant differences between the observation group and control group (10/11[90.9%] vs 5/11[45.5%], difference [95% CI]: 45.4 percentage points [11.4 to 79.4], *p* = 0.063; Fig. 6a). There were also no significant differences between the two groups at month 6 and 12(8/11[72.7%] vs 6/11[54.5%], 7/11[63.6%] vs 6/11[54.5%], respectively; difference [95% CI] 18.2 percentage points [− 21.3 to 57.7], *p* = 0.659 and 9.1 percentage points [− 31.8 to 50.0], *p* = 1.000; Fig. 6a).

### Secondary end points

#### CDAI

The improvement in CDAI scores of the observation group was better than that of the control group. There were significant differences between the groups at all follow-up time points (Table 2 and Fig. 6b).

**Table 2:** The results of simplified CDAI, PDAI, IBDQ, Wexner, and VAS scores

	**Observation group (*n*** **= 11)**	**Control group (*n*** **= 11)**	**Treatment difference (95%CI)**	***p*value**
**Simplified CDAI**				
**Baseline**	4.3 ± 0.5	4.4 ± 0.7		
**Month 3**	2.1 ± 0.3	4.9 ± 0.7		
Change from baseline	2.2 ± 0.6	0.5 ± 0.7	1.6 (1.1 to 2.2)	0.000
**Month 6**	2.5 ± 1.0	4.6 ± 0.7		
Change from baseline	1.7 ± 1.2	0.5 ± 0.7	1.3 (0.4 to 2.1)	0.006
**Month 12**	2.7 ± 1.5	4.7 ± 0.6		
Change from baseline	1.7 ± 1.4	0.5 ± 0.5	1.2 (0.2 to 2.2)	0.023
**PDAI**				
**Baseline**	8.5 ± 1.4	9.5 ± 1.7		
**Month 3**	1.3 ± 1.1	3.5 ± 0.8		
Change from baseline	7.3 ± 2.0	6.0 ± 1.3	1.3 (− 0.2 to 0.8)	0.090
**Month 6**	1.7 ± 1.6	3.5 ± 1.5		
Change from baseline	6.8 ± 1.8	6.1 ± 1.9	0.7 (− 0.9 to 2.3)	0.361
**Month 12**	1.9 ± 2.4	3.1 ± 2.1		
Change from baseline	6.6 ± 2.2	6.5 ± 2.5	0.2 (− 1.9 to 2.3)	0.858
**IBDQ**				
**Baseline**	143.5 ± 19.8	137.9 ± 29.7		
**Month 3**	202.1 ± 19.9	179.5 ± 28.0		
Change from baseline	58.6 ± 7.1	41.5 ± 36.6	17.1 (− 7.7 to 41.9)	0.158
**Month 6**	193.2 ± 31.5	174.1 ± 19.8		
Change from baseline	49.7 ± 16.1	36.5 ± 32.1	13.2 (− 9.4 to 35.8)	0.238
**Month 12**	187.8 ± 33.5	166.1 ± 23.4		
Change from baseline	44.4 ± 17.6	29.1 ± 34.6	15.3 (− 9.2 to 39.7)	0.207
**Wexner score**				
**Baseline**	1.2 ± 1.3	1.5 ± 0.7		
**Month 3**	0.8 ± 1.3	1.4 ± 0.7		
Change from baseline	0.4 ± 0.5	0.2 ± 0.4	0.1 (− 0.2 to 0.6)	0.362
**Month 6**	0.6 ± 1.0	1.2 ± 0.8		
Change from baseline	0.5 ± 0.7	0.7 ± 0.6	0.2 (− 0.8 to 0.4)	0.530
**Month 12**	0.5 ± 0.8	1.2 ± 0.8		
Change from baseline	0.6 ± 0.7	0.7 ± 0.6	0.1 (− 0.7 to 0.5)	0.750
**VAS**				
**Baseline**	0.8 ± 1.0	1.1 ± 1.0		
**Day 1**	1.5 ± 1.5	4.9 ± 0.8		
Change from baseline	0.9 ± 1.1	3.8 ± 1.0	2.9 (2.0 to 3.9)	0.000
**Day 3**	1.1 ± 1.0	4.3 ± 0.8		
Change from baseline	0.5 ± 0.9	3.2 ± 1.3	2.7 (1.7 to 3.7)	0.000
**Day7**	0.6 ± 0.5	3.2 ± 0.8		
Change from baseline	0.4 ± 0.7	2.1 ± 1.3	1.7 (0.8 to 2.6)	0.001
**Day 15**	0.4 ± 0.5	2.3 ± 0.6		
Change from baseline	0.6 ± 0.9	1.4 ± 1.1	0.7 (0.2 to 1.6)	0.112

#### PDAI

The improvement in PDAI with observation group at months 3 and 6 was greater than that with the control group, and the differences between treatments reached statistical significance (Table 2 and Fig. 6c). There were no significant differences between the groups at month 12.

#### IBDQ

After treatment, the IBDQ scores of both observation group and control group were higher than those at baseline. However, the IBDQ of the observation group improved better than that of the control group. The differences between treatments reached statistical significance at month 3, but did not reach statistical significance at month 6 and 12 (Table 2 and Fig. 6d).

#### Wexner score

The Wexner scores of all patients remained unchanged or decreased. And there were no significant differences between the groups at all follow-up time points (Table 2 and Fig. 6e).

#### VAS

In this trial, the VAS scores of the observation group was lower than that of the control group, and the differences between treatments reached statistical significance at all follow-up time points (Table 2 and Fig. 6f).

#### Inflammatory indexes

**Fig. 7:**
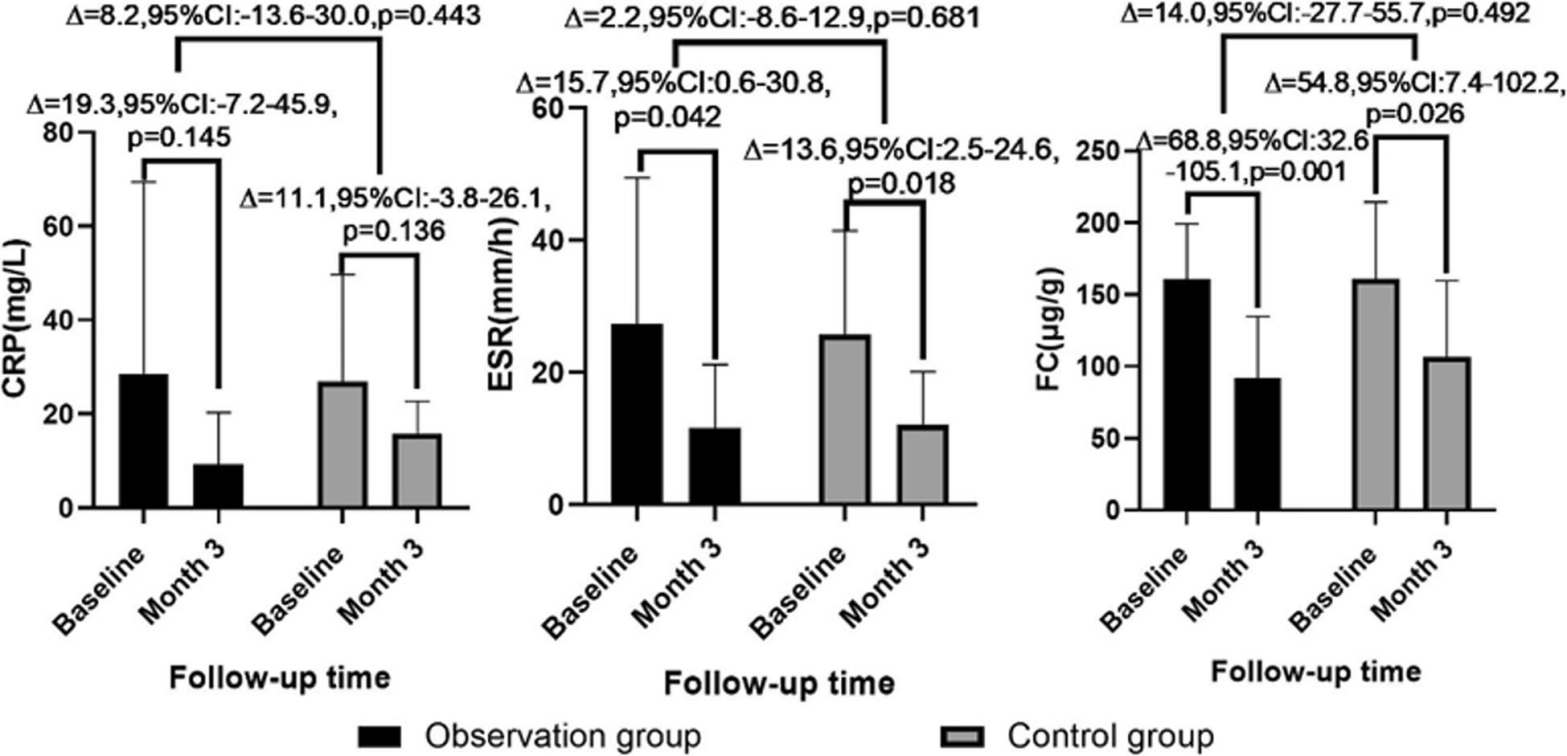
Inflammatory indexes

The inflammatory indexes decreased in both the observation group and the control group at 3 months follow-up. The relevant data were as follows: CRP 28.6 ± 40.8 VS 9.3 ± 11.1, *p* = 0.145 and 26.9 ± 22.8 VS 15.8 ± 6.9, *p* = 0.136; ESR: 27.4 ± 22.0 VS 11.6 ± 9.5, *p* = 0.042 and 25.8 ± 15.7 VS 12.2 ± 7.9, *p* = 0.018; FC 160.9 ± 38.5 VS 92.0 ± 42.9, *p* = 0.001 and 161.2 ± 53.3 VS 106.4 ± 53.3, *p* = 0.026. Compared with the baseline, there were no significant differences in the changes of CRP, ESR, and FC between the observation group and control group at month 3 (Fig. 7).

### Safety

Over the 12-month study period, there were no significant differences in the percentage of patients who experienced AEs in the observation and control groups (7/11 [63.6%] and 11/11 [100%], respectively, difference [95% CI] 36.4 percentage points [8.0 to 64.8], *p* = 0.090). The most common of which was perianal pain (Table 3).

**Table 3:** Patients with AEs up to month 12 (safety population)

**AE**	**Observation group (*n*** **= 11)**	**Control group (*n*** **= 11)**
**Overall**	7 (63.6%)	11 (100%)
**AEs**		
Pyrexia	3 (27.3%)	4 (36.4%)
Perianal pain	7 (63.6%)	11 (100%)
Anal abscess/fistula	3 (27.3%)	5 (45.5%)
Fatigue	4 (36.4%)	8 (72.7%)
**AEs leading to study withdrawal**	2 (18.2%)	3 (27.3%)
**Treatment-related AEs**	0 (0%)	11 (100%)
Perianal pain	0 (0%)	11 (100%)
Anal abscess/fistula	0 (0%)	1 (9.1%)
**Serious AEs**	0 (0%)	0 (0%)

None of patients in the observation group vs 11 of 11 (100%) in the control group experienced treatment-related AEs, and the most common of which was perianal pain. A low number of patients in both groups withdrew from the 12-month study period because they received reoperation due to AEs (observation: 2/11 [18.2%]; control: 3/11 [27.3%]). In addition, there were no serious AEs occurred in all patients. No deaths occurred during the study.

## Discussion

The guiding principle for the surgical treatment of anal fistula is to cure and reduce the recurrence rate under the premise of maximum protection of anal function [9]. Traditional surgeries inevitably damage the anal sphincter and lead to different degrees of anal incontinence [10]. Patients who undergo multiple operations have higher risk of anal incontinence and cause more complications [10, 11], as shown in Table 4.

**Table 4:** Clinical effect of traditional operation for anal fistula [10]

Surgical procedures	Recurrence rate	Anal incontinence rate
Anal fistula incision or resection	0–32%	0–31.3%
Incision-thread-drawing procedure	0–27.5%	0–22.6%
Drainage and thread drawing therapy	0–19%	0–25.7%

In order to protect the patients’ anal function, new technologies and materials are gradually applied in the treatment of anal fistulas. Reports in the literature show that the early success rate of anal-fistula plug in the treatment of low anal fistulas was 70–100%, but the late healing rate was less than 50% [12–15]. The success rate of fibrin glue was 14–63% [16, 17]. The cure rate of Mucosa advancement flap was 66–87% but the recurrence rate was high [18–20]. And mild to moderate incontinence occurred in 35% of the patients after operation. Anorectal pressure measurements showed that resting pressure and systolic pressure decreased [21]. In a retrospective analysis, Kontovounisios C et al. [22] reported 759 cases of ligation of intersphincteric fistula tract (LIFT) and found the success rate of LIFT to be 51–94%, which was not suitable for the treatment of complex anal fistulas. In conclusion, the above methods still have some shortcomings, such as low cure rate, long healing time, or high recurrence rate.

ADSC is a population of multipotent cells isolated from adipose tissue [23]. Compared with other sources of stem cells, its advantages are as follows [24–27]: ① the sources of fat are abundant, ② the patient has less pain and less damage to his donor site, ③ the isolation method of ADSC is simple and the yield is high, which is 1000 times higher than that of bone marrow stem cells, ④ ADSC has strong ability to expand in vitro and is easy to be subcultured, ⑤ ADSC has transembryonic differentiation ability, and ⑥ immune rejection of ADSC is lower than that of other stem cells. Since Garcia-Olmo et al. [28] first reported the treatment of rectovaginal fistula with adipose stem cell in 2003, this method has been a research hotspot. A randomized, double-blind, multi-center phase III clinical trial of 200 patients with complex anal fistulas in 2012 showed that ADSCs alone or in combination with fibrin glue was safe in the treatment, and the 1-year healing rate was over 50% [29]. The 52-week follow-up results of another randomized, double-blind, parallel, placebo-controlled phase III clinical trial of 212 patients with Crohn’s fistula-in-ano in 2018 showed that the combined remission rate of allogeneic ADSCs (Cx601) treatment group was significantly better than that of the placebo group, with a low recurrence rate and a low incidence of related adverse events [30]. The results of relevant researches show that whether autologous or allogeneic ADSCs are used to treat anal fistulas, they all have many advantages including no incision of normal tissue, less trauma, no sphincter injury, less pain, quick repair, low recurrence rate, and short hospitalization time. The safety and effectiveness of ADSCs have been certified, whereas other current methods of sphincterotomy and sphincter preservation surgery have not. ADSCs were approved as one of the treatments for Crohn’s fistula-in-ano by the Food and Drug Administration in 2017, and also approved by Europe in 2018.

The possible mechanisms of ADSC treatment are directional differentiation, regulation of inflammatory response, paracrine and immune regulation, and activation of angiogenesis and fibroblasts [31]. In this trial, the observation group’s closure rate of fistulas was 63.6% at month 12, and it was similar to other studies [30, 32–34]. We analyzed the situation of patients who failed in this treatment and we thought that it may be related to poor control of inflammation and the immune regulation of ADSCs except for the patient’s autoimmunity [35]. Dela Rosa et al. [36] suggested that ADSCs play an immunoregulatory role in the presence of inflammatory mediators (especially IFN-gamma) and inhibit T-lymphocyte function and regulatory T cell proliferation, resulting in the decrease of pro-inflammatory cytokines (such as IFN-gamma and TNF-alpha) and the increase of anti-inflammatory cytokines (such as IL-10). We hypothesize that inflammation in patients who failed in the ADSC treatment may not be effectively controlled, and a large amount of pro-inflammatory cytokines may still exist resulting in the nonclosing of fistulas after ADSC treatment. If inflammation could be controlled, the treatment effects could be improved.

This is the first report of using ADSCs to treat Crohn’s fistula-in-ano in the Chinese population. In this study, we used a lower concentration of ADSCs (5 × 10^6^ cells/ml) for safety reasons. This was much lower than 2–4 × 10^7^ cells/ml reported by Cao et al. [37] Therefore, the intensity of immune regulation may not have been able to completely remove infection of anal recess, possibly contributing to fistulas not closing properly. In addition, the debridement process and the basic treatment after operation still need to be refined and studied.

In this study, the short-term efficacy of ADSCs in the treatment of Crohn’s fistula-in-ano is better than that of traditional surgical procedures, and the long-term efficacy is no worse. However, this study had shown that the quality of life and anal sphincter function of patients after ADSC treatment are better than those of traditional operation. None of the patients in the study had anal incontinence due to the treatment. Safety data also showed that ADSCs were well tolerated and there was no evidence of specific adverse events associated with ADSCs, which was consistent with the results of other studies [30, 32–34].

The sample size in this study was quite limited. Large-scale randomized trials might be required to demonstrate the efficacy of ADSCs. In addition, this treatment cannot be widely promoted due to its high cost. In addition, patients have to undergo two operations, one for extraction of fat tissue, and one for injection of ADSCs. Despite the limitations mentioned above, our research shows that this treatment is safe and convenient, does not cause undue damage of normal tissue, and provides good tolerance, repeatability, quick recovery, less pain, less damage to anal function, and high quality of life during perioperative period.

## Conclusion

The practice of surgical treatment has evolved from destructive excision to repair and has now developed to reconstruction and regeneration. ADSCs for Crohn’s fistula-in-ano reflect the scientific concept and trend of reconstruction and regeneration. In conclusion, as a new technology which accords with the principle of anal fistula treatment, it is an effective method to treat Crohn’s fistula-in-ano.

## Data Availability

The datasets used and/or analyzed during the current study are available from the corresponding author upon request.

## Abbreviations

CD: Crohn’s disease
ADSC: Adipose-derived stem cell
IBD: Inflammatory bowel disease
CDAI: Crohn’s Disease Activity Index
SVF: Stromal vascular fraction
MRI: Magnetic resonance imaging
ERUS: Endorectal ultrasonography
PDAI: Perianal Disease Activity Index
IBDQ: Inflammatory Bowel Disease Questionnaire
VAS: Visual analog score
CRP: C-reactive protein
ESR: Erythrocyte sedimentation rate
FC: Fecal calprotectin
AE: Adverse event
LOCF: Last observation carried forward
LIFT: Ligation of intersphincteric fistula tract
